# Editorial: Resistance to Endocrine Therapies in Cancer

**DOI:** 10.3389/fendo.2020.00196

**Published:** 2020-04-09

**Authors:** Prathibha Ranganathan, Anindita Chakrabarty, Stephen Hiscox, Anil Mukund Limaye, Veronica Vella

**Affiliations:** ^1^Centre for Human Genetics, Bengaluru, India; ^2^Department of Life Sciences, Shiv Nadar University, Greater Noida, India; ^3^School of Pharmacy and Pharmaceutical Sciences, Cardiff University, Cardiff, United Kingdom; ^4^Department of Biosciences and Bioengineering, Indian Institute of Technology Guwahati, Guwahati, India; ^5^Department of Clinical and Experimental Medicine, University of Catania, Garibaldi-Nesima Hospital, Catania, Italy

**Keywords:** endocrine therapy, growth factor signaling, tumor microenvironment, non-canonical signaling, drug resistance, hormone ablation, estrogen receptor

Therapeutic resistance in cancer is one of the major and persistent problems in disease management. It is a major challenge for radiotherapy as well as both targeted and non-targeted chemotherapies. Understanding of the underlying genetic, epigenetic and signaling mechanisms, both within the tumor (intrinsic) as well as in the tumor microenvironment (extrinsic) would facilitate development of better disease management strategies.

Endocrine therapy refers to a group of chemotherapies which are targeted to disrupt hormonal signaling pathways. The most commonly targeted pathways are the estrogen and androgen signaling pathways, which have major roles to play in the cancers of breast and prostate, respectively. Breast cancer is the second most common cancer among women and prostate the second among men^1^. Both these organs and also the cancers originating in these organs are heavily dependent on the steroid hormones for their growth, survival and proliferation. Hence, the obvious choice of first line of therapy for these cancers is to disrupt the steroid hormone synthesis and action, i.e., endocrine therapy. Although initially very effective, a majority of the hormone-dependent cancers develop resistance to hormonal ablation and evolve into a hormone-independent phenotype, rendering the endocrine therapies ineffective. Once this refractoriness sets in, the cancers become very aggressive and difficult to treat. Whether it is primary or adaptive/acquired resistance to endocrine therapy, it remains a yet unsolved clinical challenge.

For the last several years, attempts have been made to understand the mechanisms responsible for resistance to endocrine therapies with the aim of developing more effective and less toxic treatment strategies. Among the relatively well-understood mechanisms of endocrine therapy resistance are *de novo* mutations in the hormone receptor genes such as *ESR* 1 and *ESR* 2, although seen in a small percentage of cases. Differential activation of signaling pathways such as PI3K/Akt and Ras-MAPK is seen more often in tumors with acquired resistance. Aberrations in cell cycle regulators such as Cyclin D1, RB, or MYC and activation of receptor tyrosine kinases (examples ErbB2, FGFR, IR, IGF1R) have also been implicated in development of endocrine therapy resistance [reviewed in (1)]. More recently, alterations in autophagy (2), metabolism (3), etc. have been associated with acquisition of endocrine-therapy resistance.

This collection of articles attempts to provide a comprehensive picture of the challenges faced due to emergence of resistance to endocrine therapies in breast cancer and an insight into the current understanding of the mechanisms underlying the same. The article collection includes comprehensive reviews on the role of mutations in estrogen receptors and the role of alternative signaling pathways such as PI3K/Akt, mTOR etc. in the development of endocrine resistance in breast cancer (Rani et al.; Haque and Desai). Over the last decade, the role of the tumor niche in metastasis, relapse, and therapeutic resistance has been gaining a lot of importance. An article by Dr. Simian's group gives an overview of the various microenvironment components and their influence on endocrine therapy in breast cancer (Diaz Bessone et al.). Another article by Dr. Ranganathan's group gives an insight into the role of non-canonical estrogen receptors in conferring endocrine resistance (Ranganathan et al.). Despite comprehensive coverage of current status of research in these areas, some aspects such as the role of autophagy, the effect of changes in the organization of the 3D genome, etc. on endocrine resistance would be important to gain a better understanding of the phenomenon and in developing better strategies for treatment. This endeavor has brought together different research groups and we hope that this can lead to improved collaborative research.

## Author Contributions

PR co-ordinated the Research Topic and the editorial. AC, SH, AL, and VV contributed for the development of the Research Topic, suggested, and invited the participants and also helped in the peer review process.

### Conflict of Interest

The authors declare that the research was conducted in the absence of any commercial or financial relationships that could be construed as a potential conflict of interest.

## Footnotes

^1^Available online at: www.cancer.org.

## Abbreviations

PI3K: Phosphatidylinositol 3-kinase
Akt, Alias Protein Kinase B: a serine/threonine kinase
MAPK: Mitogen Activated Protein Kinase
RB: Retinoblastoma
ErbB2, A receptor tyrosine kinase: also known as HER2 (from human epidermal growth factor receptor 2)
FGFR: Fibroblast Growth Factor Receptor
IR: Insulin Receptor
IGF1R: Insulin Like Growth Factor 1 Receptor
mTOR: Mammalian Target of Rapamycin
3D genome: Three Dimensional Genome
*ESR*: Gene coding for estrogen receptor.
